# Awareness towards stroke among high school students in Brazil: a cross-sectional study

**DOI:** 10.1590/1516-3180.2021.0659.R2.13102021

**Published:** 2022-05-02

**Authors:** Mateus de Sousa Rodrigues, Leonardo Fernandes e Santana, Alanderson Passos Fernandes Castro, Karyne Krysley Almeida Coelho, Manoel Pereira Guimarães, Orlando Vieira Gomes, Paulo Adriano Schwingel, Renato Bispo de Cerqueira, Marcos Duarte Guimarães, José Carlos de Moura

**Affiliations:** I Undergraduate Medical Student, Universidade Federal do Vale do São Francisco (UNIVASF), Petrolina (PE), Brazil; 2 Undergraduate Medical Student, Universidade Federal do Vale do São Francisco (UNIVASF), Petrolina (PE), Brazil; 3 Undergraduate Medical Student, Universidade Federal do Vale do São Francisco (UNIVASF), Petrolina (PE), Brazil; 4 Undergraduate Medical Student, Universidade Federal do Vale do São Francisco (UNIVASF), Petrolina (PE), Brazil; 5 Undergraduate Medical Student, Universidade Federal do Vale do São Francisco (UNIVASF), Petrolina (PE), Brazil; 6 MSc. Professor, School of Medicine, Universidade Federal do Vale do São Francisco (UNIVASF), Petrolina (PE), Brazil; 7 PhD. Professor, Human Performance Research Laboratory, Universidade de Pernambuco (UPE), Pernambuco (PE), Brazil; 8 MD. Professor, School of Medicine, Universidade Federal do Vale do São Francisco (UNIVASF), Petrolina (PE), Brazil; 9 PhD. Professor, School of Medicine, Universidade Federal do Vale do São Francisco (UNIVASF), Petrolina (PE), Brazil; 10 PhD. Professor, School of Medicine, Universidade Federal do Vale do São Francisco (UNIVASF), Petrolina (PE), Brazil

**Keywords:** Stroke, Risk factors, Education, Awareness, Surveys and questionnaires, Cerebrovascular accident, Self-structured questionnaire survey, Situation awareness

## Abstract

**BACKGROUND:**

Stroke is one of the main causes of death worldwide. Educational interventions on stroke are potentially effective in reducing the period between the onset of symptoms and the initial emergency medical assistance.

**OBJECTIVES:**

To assess high school students’ knowledge of stroke.

**DESIGN AND SETTING:**

Cross-sectional study conducted in high schools in northeastern Brazil.

**METHODS:**

A self-structured questionnaire survey regarding stroke awareness was applied among high school students in northeastern Brazil. Data were collected between 2018 and 2019. The chi-square test and other descriptive statistics were used. Univariate and multivariate analyses were performed using logistic regression.

**RESULTS:**

A total of 1,788 students were analyzed. Eighty percent (n = 1430) of them did not have the minimum knowledge on how to act in a stroke situation. Only 10% (n = 179) presented the ideal knowledge on how to act. Males presented lower levels of knowledge on risk factors (odds ratio, OR: 0.62%; 95% confidence interval, CI: 0.49-0.79) and signs and symptoms of stroke (OR: 0.63%; 95% CI: 0.52-0.77). Students with ≥ 10 years of schooling (OR: 1.64%; 95% CI: 1.30-2.07) demonstrated greater knowledge of signs and symptoms of stroke. Students aged 18 years (OR: 1.70%; 95% CI: 1.14-2.52) demonstrated greater knowledge than other ages regarding the telephone number of the emergency medical services.

**CONCLUSIONS:**

There was a knowledge deficit with regard to recognizing stroke and activating the emergency medical services. The findings apply to the sample investigated and suggest that there is a need for stroke educational interventions, starting in high school.

## INTRODUCTION

Stroke is one of the main causes of morbidity and mortality worldwide.^1^ In addition to affecting individuals, it also requires significant expenditure from healthcare budgets.

Developing countries, such as Brazil, are in epidemiological transition, characterized by a drop in the incidence of infectious diseases and, at the same time, an accumulation of modifiable and non-modifiable risk factors for chronic non-communicable diseases (CNCDs).^2^ Analogously, CNCDs are risk factors for stroke and other vasculopathies, and it may therefore be foreseen that aging of the population will be accompanied by significantly increased incidence of stroke.^3^

Educational measures regarding stroke are potentially effective in reducing the critical period between the onset of symptoms and the initial emergency medical assistance.^4^ Early recognition of stroke is of utmost importance because it can modify the natural course of the disease by enabling specific treatment strategies in its early stages. Based on this assumption, it is therefore imperative that the population should possess knowledge concerning stroke; be able to recognize the risk factors, signs and symptoms; and be aware of preventive measures for this condition, as well as the correct conduct when faced with a stroke.^5^

With this in mind, it can be understood that this knowledge is important for the young population, since many of these individuals, after leaving high school, will take up jobs in places with large gatherings of people, such as shopping malls, airports or parks. They should therefore be able to recognize the signs and symptoms of stroke, so that the emergency services can be called. This scenario should therefore be seen as an ideal substrate for dissemination of preventive and health promotion measures, in order to minimize the negative impact of this nosological entity.

## OBJECTIVE

The aim of the present study was to assess the knowledge of high school students about stroke.

## METHODS

This was a cross-sectional study conducted in five municipalities in northeastern Brazil (Petrolina, Afrânio, Dormentes, Salgueiro and Cedro) between August 2018 and July 2019. Volunteer high school students agreed to participate in the study and respond to the data collection instrument in the form of a questionnaire. The following inclusion criteria were adopted: (I) aged between 15 and 18 years and (II) attending a public high school. Those who did not complete the entire questionnaire were excluded.

The questionnaire was prepared specifically for this study and was based on a review of the literature encompassing other studies that had also assessed the level of knowledge regarding stroke in their respective target audiences. Each participant was required to answer the following questions: 1) Do you know what a stroke is? 2) Can you indicate at least three signs or symptoms of a stroke? 3) Can you indicate at least three risk factors for a stroke? 4) What would you do in a stroke situation? and 5) What is the telephone number of the emergency medical services (Serviço de Atendimento Móvel de Urgência, SAMU) in Brazil? Sociodemographic data such as age, sex and schooling were also collected.

In accordance with the definition used in the present study, knowledge of stroke was assessed based on the ability to make decisions when faced with a stroke situation. It was classified into three levels: a) ideal (able to recognize three symptoms and three risk factors, and knowing how to activate the emergency medical services); b) minimum required (able to recognize one symptom and one risk factor, and knowing how to activate the emergency medical services); and c) below the minimum (not meeting any of the abovementioned characteristics).

Thus, respecting the ethical principles of the Declaration of Helsinki (1964), ethical approval was obtained from the Universidade Federal do Vale do São Francisco (UNIVASF); under protocol No. 3.609.473; date: September 30, 2019. During the survey, no subject consent was required because no identifiable data were collected. The final survey instrument contained eight items (**Annex 1**).

## RESULTS

A total of 1,870 questionnaires were obtained, of which 82 were excluded due to insufficient data. Thus, the final analysis included a spectrum of 1788 students, of whom 982 (54.9%) were female and 806 were male (45.1%), as presented in **Table 1**. The participants were aged between 15 and 18 years. There were 882 students (49.3%) with 10 years of schooling; 538 students (30.1%) with 11 years; and 368 students (20.6%) with 12 years (**Table 1**).

**Table 1:** Demographics and stroke knowledge mentioned by participants (n = 1,788)

Variables	n	%
**Sex**		
Male	806	45.1
Female	982	54.9
**Years of schooling**		
10	882	49.3
11	538	30.1
12	368	20.6
**I know what stroke is**		
Yes	1,368	76.5
No	420	23.5
**I know some signs and symptoms for stroke**		
None	1,047	58.6
1	237	13.3
2	254	14.2
≥ 3	250	14.0
**I know some risk factors for stroke**		
None	1,413	79.0
1	97	5.4
2	84	4.7
≥ 3	194	10.9
**I know the emergency medical services telephone number**		
Yes	1,262	70.6
No	526	29.4

The Brazilian acronym for stroke (“AVC”) was recognized by 77.8% (n = 1,368) of the participants (**Table 1**). However, 58.6% (n = 1,047) of the students did not know any signs and symptoms of stroke. Only 14.0% (n = 250) of the participants knew three or more signs and symptoms of stroke, and 79.0% (n = 1,413) were unaware of the risk factors for the disease. Only 10.9% (n = 194) knew three or more risk factors for stroke. A total of 70.6% (n = 1,262) of the students were unaware of the telephone number of the emergency medical services (192) (**Table 1**).

**Figure 1.**
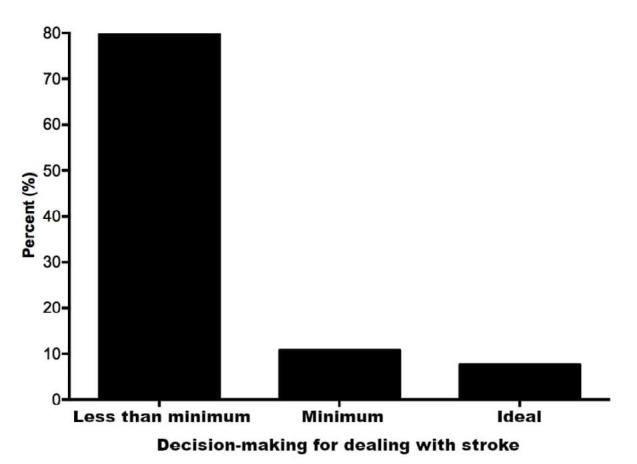
Decision-making capacity for dealing with stroke.

With regard to the ability to act in the event of a stroke, 80% of the students interviewed did not have the minimum knowledge on how to proceed when faced with this event, and only 10% demonstrated that they had the ideal knowledge on how to act, as presented in **Figure 1**.

Based on the odds ratio calculated for the association between basic characteristics and knowledge of stroke, males presented lower knowledge regarding risk factors (odds ratio, OR: 0.62%; 95% confidence interval, CI: 0.49-0.79) and signs and symptoms of stroke (OR: 0.63%; 95% CI: 0.52-0.77). Students with more than 10 years of schooling (OR: 1.64%; 95% CI: 1.30-2.07) presented greater knowledge of the signs and symptoms of stroke. Students aged 18 years (OR: 1.70%; 95% CI: 1.14-2.52) knew the emergency medical services telephone number more often than those of other ages (**Table 2**).

**Table 2:** Odds ratios of the association between baseline characteristics and stroke knowledge

		Knowledge of at least one stroke symptom or risk factor		
Variables	n (%)	Stroke symptoms	Stroke risk factors	EMS telephone
		OR (95% CI)	OR (95% CI)	OR (95% CI)
**Sex**				
Female	982 (54.9)	1	1	1
Male	806 (45.1)	0.63 (0.52-0.77)	0.62 (0.49-0.79)	1.04 (0.85-1.28)
**Schooling (years)**				
10	882 (49.3)	1	1	1
> 10	906 (50.7)	1.64 (1.30-2.07)	1.27 (0.96-1.68)	1.19 (0.93-1.52)
**Age (years)**				
15	427 (23.9)	1	1	1
16	697 (39.0)	1.29 (0.83-2.00)	0.86 (0.63-1.18)	1.04 (0.66-1.65)
17	486 (27.2)	1.02 (0.68-1.51)	0.88(0.60-1.28)	1.21 (0.81-1.81)
18	178 (9.9)	0.98 (0.68-1.40)	1.19(0.74-1.91)	1.70 (1.14-2.52)

OR = odds ratio; CI confidence interval; EMS = emergency medical services.

## DISCUSSION

This study established that only 10% of the population studied (high school students) were aware of how to act correctly in the event of a stroke. This demonstrates a worrying reality concerning deficiencies in the basic training of students in terms of the concept of first aid.

However, this observed deficiency is not exclusive to this study, since these data are in agreement with previous studies. Thus, it was observed that only 14% of adults in the state of Michigan would take the right attitude when faced with this situation.^6^ Within the same perspective, the results presented in **Figure 1** demonstrate that 80% of high school students in Brazilian public schools did not present even the minimum conditions to be able to assist an individual in the event of a stroke situation, and 79.5% of these students were unaware of any risk factors regarding this condition (**Table 1**).

Other studies, in which it was shown that the prognosis for stroke victims who receive early care was better, have demonstrated that the costs resulting from treatment of stroke cases are lower when patients are attended early.^7,8^ Early arrival of patients at medical services depends on prior knowledge of stroke symptoms. One strategy for increasing the population’s awareness about stroke symptoms is to provide training on stroke recognition, starting from the time of high school education. Planning for this could be facilitated by means of investments in first aid education. Through this learning, students would be better prepared to activate the emergency services in a timely manner.^7,8^ The present study evaluated the level of knowledge of high school students about stroke and can guide educational policies regarding the subject, and especially regarding the deficit in recognizing stroke and activating the emergency medical services.

The students’ lack of knowledge regarding first aid for stroke may reflect a lack of interest in the topic and an absence of infrastructure in educational institutions that focuses on theoretical and practical training relating to strokes. This lack of information results from the fact that this topic is rarely addressed in schools. Recent studies conducted outside of Brazil have indicated that there is a need for a broad educational program on stroke within the school context.^9^ Moreover, Brazilian students are not trained for this because educators are unprepared in relation to basic first aid measures in emergency situations.^10^

Investment in education is a potential solution for mitigating the low preparedness of Brazilian students in the event of a stroke and for providing training for educators and students. Seminars and posters are among the teaching strategies that have been recommended by a number of authors.^11^ Studies conducted in countries such as the United States, Portugal and South Korea have demonstrated that stroke intervention taught in schools is an effective alternative for making students aware of the risk factors, symptoms and management of stroke patients.^12,13^ Additionally, this approach in educational institutions has also increased the knowledge of students’ parents.^11^ Dissemination of knowledge acquired within the school scenario tends to provide a long-term contribution to the knowledge of the population, since parents may also disseminate this knowledge. Furthermore, this knowledge may also be used in the job market, since upon leaving school, many young people choose to enter the job market working in places with large gatherings of people, such as shopping malls, parks and airports, where the chances of witnessing a stroke are greater.

The lack of knowledge of high school students in Brazilian schools regarding stroke, along with their inability to activate the emergency services, may cause complications for patients. The time that elapses until hospital admission, among individuals with acute stroke, is an important determinant of the prognosis. The ideal, for patients to receive care with the greatest chance of success, is that admission should not be more than 4.5 hours after the onset of the first symptoms of the most prevalent type of stroke, i.e. the ischemic subtype.^14^ Delaying patient admission to a hospital will considerably reduce the chances of being able to treat complications, considering that studies have reported that if patients are not treated during an ischemic stroke, they may lose 1.9 million neurons every minute.^15^

In reality, few patients arrive at emergency units in a timely manner and, therefore, patients are not always able to receive the initial treatment.^16,17^ The main reason why stroke patients experience delays in arriving at a hospital relates to the lack of knowledge on this subject.^17^ Analysis on the situation of students in Brazilian high schools from the results presented here shows that most of the target population of this study constituted a group presenting characteristics that are likely to cause delay in the admission of patients with stroke, i.e. this was a group with little understanding of the subject.

Students with more than 10 years of schooling (OR: 1.64%; 95% CI: 1.30-2.07) had greater knowledge of the signs and symptoms of stroke (**Table 3**). This result is similar to what had been observed in other studies developed in the Brazilian population, in which individuals with higher levels of education had greater ability to recognize the signs and symptoms of stroke.^18,19^ The group of 18-year-old students (OR: 1.70%; 95% CI: 1.14-2.52) knew the emergency medical services telephone number more often than did other age groups (**Table 2**). This pattern was probably due to the fact that the longer a person lives, the greater the chances will be that this person will witness a situation in which there is a need to call the emergency medical services, or will watch a newscast containing information on the emergency medical services telephone number or will study this information when taking part in a public job or educational entrance competition. In Brazil, young people aged 18 years intensify their studies with a view to entering college/university and/or the labor market. On the other hand, the difficulties in recognizing stroke were more evident among males.

Males demonstrated less knowledge with regard to risk factors (OR: 0.62%; 95% CI: 0.49-0.79) and signs and symptoms of stroke (OR: 0.63%; 95% CI: 0.52-0.77), as presented in **Table 2**. In general terms, there was a tendency for female adolescents to be more interested in a greater number of health-related topics than were male adolescents.^20^ Population studies carried out in Brazil have also reported that there was greater lack of knowledge among men, in terms of both risk factors and the signs and symptoms of stroke.^18,19^

Development of the present study has reinforced the hypothesis that greater inability to recognize stroke is a trend that is perpetuated from adolescence onwards. Thus, it was observed in this study that, like in developed countries such as the United States,^11^ Brazilian high school students also seem to share similar difficulties in recognizing stroke. This highlights the importance of consolidating knowledge on health-related topics even during high school. Previous studies have reported that knowledge of good practices in healthcare that is consolidated during adolescence tends to last throughout life.^18^

Nonetheless, although the present study provides a useful panorama to guide educational and public health policies, it is important to note that these findings reflect the reality of five municipalities in northeastern Brazil.

## CONCLUSIONS

Although stroke is among the major causes of morbidity and mortality in the world, a considerable deficit of knowledge was observed with regard to recognizing the clinical features of stroke, particularly among males, and to activating the emergency services. The data presented here, from the sample investigated, are alarming, since such knowledge is essential for reducing the interval between the onset of symptoms and instituting the first therapeutic measures, which is a determining factor for the prognosis of stroke victims.

## Annex 1

**Table:** English version of the questionnaire.

Questions on stroke knowledge:
1. Age:
2. Sex: F_ M_
3. Years of schooling:
4. Do you know what a stroke is?
5. Can you indicate at least three signs or symptoms of a stroke?
6. Can you indicate at least three risk factors for a stroke?
7. What would you do in a stroke situation?
8. What is the telephone number of the emergency medical services in Brazil?
